# Applications of ChatGPT in Heart Failure Prevention, Diagnosis, Management, and Research: A Narrative Review

**DOI:** 10.3390/diagnostics14212393

**Published:** 2024-10-27

**Authors:** Sai Nikhila Ghanta, Subhi J. Al’Aref, Anuradha Lala-Trinidade, Girish N. Nadkarni, Sarju Ganatra, Sourbha S. Dani, Jawahar L. Mehta

**Affiliations:** 1 Department of Internal Medicine, University of Arkansas for Medical Sciences, Little Rock, AR 72205, USA; ghantasain@uams.edu; 2 Division of Cardiology, University of Arkansas for Medical Sciences, Little Rock, AR 72205, USA; sjalaref@uams.edu; 3 Division of Cardiology, Ichan School of Medicine at Mount Sinai, New York, NY 10029, USA; anu.lala@mountsinai.org (A.L.-T.); girish.nadkarni@mountsinai.org (G.N.N.); 4 Division of Cardiology, Lahey Hospital and Medical Center, Burlington, MA 01805, USA; sarju.ganatra@lahey.org

**Keywords:** heart failure, large language models, artificial intelligence, machine learning, ChatGPT, natural language processing

## Abstract

Heart failure (HF) is a leading cause of mortality, morbidity, and financial burden worldwide. The emergence of advanced artificial intelligence (AI) technologies, particularly Generative Pre-trained Transformer (GPT) systems, presents new opportunities to enhance HF management. In this review, we identified and examined existing studies on the use of ChatGPT in HF care by searching multiple medical databases (PubMed, Google Scholar, Medline, and Scopus). We assessed the role of ChatGPT in HF prevention, diagnosis, and management, focusing on its influence on clinical decision-making and patient education. However, ChatGPT faces limited training data, inherent biases, and ethical issues that hinder its widespread clinical adoption. We review these limitations and highlight the need for improved training approaches, greater model transparency, and robust regulatory compliance. Additionally, we explore the effectiveness of ChatGPT in managing HF, particularly in reducing hospital readmissions and improving patient outcomes with customized treatment plans while addressing social determinants of health (SDoH). In this review, we aim to provide healthcare professionals and policymakers with an in-depth understanding of ChatGPT’s potential and constraints within the realm of HF care.

## 1. Introduction

Heart failure (HF) represents an increasingly prevalent clinical condition, impacting more than 6.7 million individuals in the United States alone [1]. Projections indicate a 46% increase in prevalence, with more than 8 million people expected to be affected by 2030 [2]. Despite the advancements in understanding the pathophysiology and management, HF imposes a significant financial burden on the US healthcare system, totaling approximately USD 43 billion in expenditure in 2020 [2]. Notably, 80% of these medical costs stem from HF-related hospitalizations [3]. HF hospitalizations also have a considerably high readmission rate, with one in every five patients getting readmitted every 30 days and every second patient being hospitalized in 6 months [4,5]. Guideline-directed medical therapy (GDMT), combined with closer follow-up, has improved HF-related mortality and mitigated readmissions [6,7]. However, the paucity of medical resources and workforce emphasizes the pressing need for innovative approaches to curtail HF-related rehospitalizations [8]. Artificial intelligence (AI) and machine learning (ML) techniques, with the ability to integrate large datasets, have been explored as potential tools in this regard [9]. ML models have demonstrated proven benefits when studied alongside conventional statistics in various fields of cardiovascular medicine [10]. AI algorithms have demonstrated the potential to improve HF care by supporting clinical decision-making, optimizing treatment allocation to highest-risk patients or identifying those who benefit most from therapy, predicting adverse outcomes and ability to detect patients with sub-clinical disease or worsening HF [9,11]. ML models have also been shown to enhance HF diagnosis by analyzing a wide range of data from various sources such as electrocardiograms, echocardiography, remote monitoring devices, and heart sounds [12,13]. Recently, ChatGPT (Generative Pre-trained Transformer), the state-of-the-art conversational model, has attracted worldwide attention for its capability of generating human-like responses to natural language inputs [14]. As an integral part of open AI’s pre-training transformer models, it currently represents one of the most extensively accessible language models [15]. With the ability to understand and replicate intricacies and nuances of human language, ChatGPT is rapidly emerging as a potentially revolutionary tool in practicing modern-day medicine [16,17]. The language model has proven effective in assisting physicians in clinical decision-making and formulating personalized therapeutic strategies [16,18,19]. ChatGPT can help fill the gaps in HF literature by synthesizing extensive datasets, offering concise summaries of recent research, and identifying inconsistencies within clinical guidelines. ChatGPT language model aids in knowledge dissemination by providing accessible explanations of complex topics, potentially enhancing patient–clinician communication and bridging the knowledge gap. In addition, contrary to conventional databases, it generates real-time, context-specific responses, facilitating decision support and expediting research synthesis. Its interactive nature, coupled with the potential for continuous refinement through feedback, positions it to eventually offer more precise, evidence-based recommendations that can complement clinical decision-making in HF management. In this comprehensive review, we introduce the concept of utilizing ChatGPT algorithms in preventing and managing HF, including aspects of device therapy and heart transplantation. Finally, we outline the existing limitations in adopting ChatGPT technology regarding ethical considerations and discuss practical solutions.

## 2. Introduction to ChatGPT Language Models

**Figure 1:**
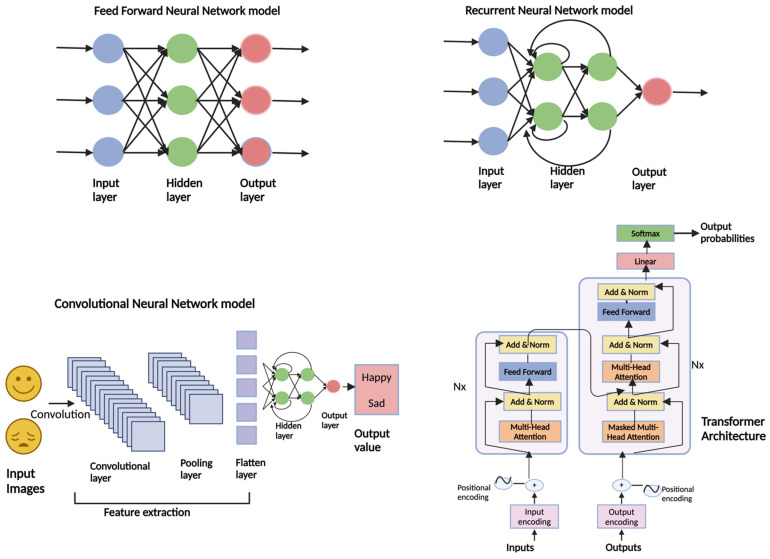
Schematic explanation of various neural network models. The Feed Forward Neural Network model is depicted with a linear arrangement of input, hidden, and output layers, illustrating a straightforward data processing path without feedback connections. The Recurrent Neural Network model handles sequential data via recurrent connections in the hidden layers, allowing for temporal data processing, shown with output probabilities. The Convolutional Neural Network model is detailed with layers designated for feature extraction from images, including convolutional, pooling, and flattened layers, leading to an output that classifies emotional expressions. Lastly, the Transformer Architecture uses components such as Multi-Head Attention and positional encodings to manage dependencies in input data effectively, exemplifying its advanced data processing capabilities.

**Figure 2:**
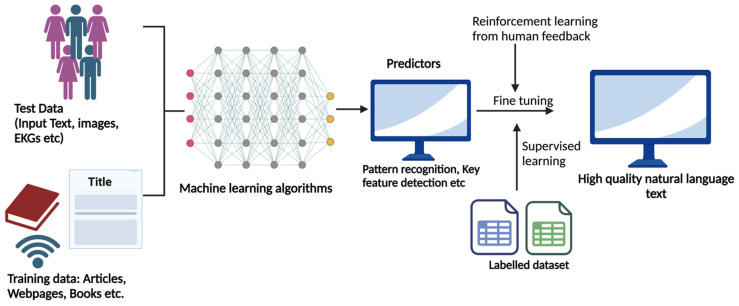
ChatGPT model workflow explained from a deep learning perspective. Test data, including text, images, and electrocardiograms (ECG), are processed through layered machine learning algorithms, producing predictors via pattern recognition. These predictors are refined through reinforcement learning from human feedback and supervised learning using a labeled dataset, enhancing the model’s natural language generation capabilities.

The rapid advancement of AI and natural language processing (NLP) have led to the development of sophisticated Large Language Models (LLMs), such as open AI’s GPT series [20,21] These models use deep learning techniques operating through neural networks of multiple nodes arranged in interconnected layers. Upon receiving data inputs, specific neurons in the input layer become activated, initiating a cascade of commands through hidden layers until reaching the output layer. The complexity of command increases as the information moves through successive hierarchical layers. Deep learning includes various types of neural networks, each optimized for distinct data types and tasks. For example, while traditional AI/ML models often overlook the temporal dimension in data analysis, certain specialized neural networks such as recurrent neural networks (RNNs) and convolutional neural networks (CNNs) excel in this area [12]. RNNs are designed to handle sequential data, such as speech, by preserving input memory. At the same time, CNNs are effective for grid-like data like images by applying convolutional filters that maintain relationships between elements [22]. However, RNNs and CNNs perform sub-optimally with diminishing accuracy with larger data due to their sequential processing limiting the possibility of parallel computation [22]. To improve this, Vaswani et al., in 2017, introduced the transformer architecture model, which relies on self-attention mechanisms to process input data in parallel [23]. The self-attention mechanism enables the transformer model to process each word by assessing its importance relative to every other word. The mechanism works by creating three vectors for each word: Query (Q), Key (K), and Value (V). The model calculates attention scores by taking the dot product of Q and K vectors, which are then scaled and normalized using a softmax function to produce attention weights. These weights help the model focus on each word, allowing it to capture dependencies regardless of their position in the sequence. Multiple sets of Q, K, and V vectors (known as attention heads) simultaneously capture different aspects of word relationships. As a result, transformers can be particularly effective in handling large-scale sequential tasks like language translation and text summarization (Figure 1) [23]. This technology paved the way for Generative Pre-trained Transformers (GPTs) and highly efficient and scalable language models. Open AI’s initial work on language models started with GPT-1 and GPT-2, each with an increasing number of parameters, eventually leading to GPT-4 with 1.76 trillion parameters [14,20,24]. ChatGPT, a freely accessible chatbot that can generate new, original content, often in text, images, audio, and more, is based on the GPT-3.5 architecture [17]. ChatGPT-4.0, based on GPT-4 architecture, is an advancement and was trained on a more extensive and more diverse dataset, which includes more recent data up to 2023, allowing for improved understanding and generating capabilities [21]. The critical feature of ChatGPT models lies in their ability to pre-train on a large corpus of text data, including textbooks, websites, articles, etc., in an unsupervised way [25]. This pre-training allows the model to predict patterns and follow words in a sentence based on previous words. After pre-training, the model is subjected to fine-tuning on specific downstream tasks such as text classification, language translation, question-answering, etc. For example, the model can be fine-tuned for sentimental analysis (e.g., positive, negative, and neutral) using a labeled dataset, and, once fine-tuned, the model can be effective in analyzing sentiment in new, unseen text. The ChatGPT model workflow is depicted in Figure 2, and milestones toward the evolution of ChatGPT are shown in Table 1.

## 3. Role of ChatGPT in HF Prevention

The pathophysiology of HF stems from multifaceted etiologies with complex interlinking among numerous risk factors. Risk factors associated with HF onset and progression include genetic disposition, lifestyle, socioeconomic factors, co-morbidities, medication use, compliance, laboratory and imaging features, and serum biomarkers [26,27]. Early identification of risk factors is essential, given the impact of mortality and morbidity associated with HF prevalence [28]. In this context, we elucidate several clinical instances in which ChatGPT can assist in HF prevention.

Risk Assessment: The generative AI model, when prompted, can help an individual evaluate their demographics, lifestyle, family history, and co-morbidities. Such information can provide insights into risk stratification and help clinicians offer personalized recommendations tailored to each individual’s risk profile, thus aiding clinicians in precision medicine practices. Sarraju et al. conducted an explorative study evaluating the appropriateness of AI model responses to simple cardiovascular preventive questions. They identified that the ChatGPT-3.5 model generated appropriate responses for 21 out of 25 questions (84%), as determined by preventive cardiologists [29]. The study supports the potential of generative AI in providing appropriate and helpful information for cardiovascular disease prevention, which can be integrated into clinical practices to improve patient care and risk assessment.

Health promotion: Besides prediction and diagnosis, the ChatGPT model can promote health awareness through education and guidance via engaging tools, such as having a virtual chat assistant depicting the user’s exercise goals and healthy dietary habits via icons or flow charts [30].

## 4. Role of ChatGPT in HF Diagnosis

The clinical spectrum of HF is divided into three groups based on left ventricular ejection fraction (LVEF): reduced (HFrEF), preserved (HFpEF), and mildly reduced (HFmrEF) with EF ranges <40%, ≥50%, and 41–49%, respectively, and four stages based on symptoms: Stage A (At risk for HF), stage B (Pre-HF), stage C (symptomatic HF), and stage D (advanced HF) [6,31]. Accurate diagnosis of HF is critical and relies heavily on early identification of symptoms/signs and risk factors. However, current evidence suggests that unfavorable outcomes can still occur even in the early stages of HF or among those who achieve recovery under optimized treatment [32]. These observations emphasize the need for novel and more objective diagnostic criteria covering the entire spectrum of HF. ChatGPT can advance HF diagnosis by analyzing the large spectrum of patient data and leveraging its natural language processing and ML capabilities. We provide various instances where ChatGPT can offer multiple avenues to improve HF diagnosis [33,34].

Education and awareness: ChatGPT can create informative articles or videos and raise public awareness by explaining HF risk factors and symptomatology. Utilizing ChatGPT to disseminate information on HF presents a notable advancement over traditional educational practices, as ChatGPT offers unparalleled accessibility, quickly reaching a broader audience through digital means. It provides personalized, real-time interactions that adapt to individual user queries, enhancing relevance and engagement compared to static educational materials. Its cost-effectiveness and scalability make it a viable option for extensive public health campaigns, capable of simultaneously handling vast volumes of inquiries without additional resources. Kasab et al. evaluated the ability of the ChatGPT-3.5 model and found that the language model offered largely accurate (88%) patient-facing recommendations for managing hypertension in adults [33]. ChatGPT also performed well in answering questions on hypertension treatment across age, gender, and different ethnicities. The study highlights the potential of ChatGPT in assisting individuals in developing a deeper understanding of their disease condition and improving patients’ experience and communication [33].

Symptom analysis and real-time data collection: For example, when a patient reports chest pain, shortness of breath, or lower extremity edema to the ChatGPT chatbot, the language model can dynamically gather and analyze patient-reported symptoms, assessing their severity in real-time and highlighting the red flags. Such a process could help individuals identify potential problems and encourage them to seek immediate medical attention. For example, Harskamp et al. designed a proof-of-concept AMSTELHEART-2 study to interpret symptoms and manage common cardiac conditions. They found that the ChatGPT-3.5 model answered straightforward, low-complex patient-to-physician questions related to HF with 90% accuracy [34]. However, the study showed limitations in more complex scenarios, matching expert opinion only 50% of the time. This study suggests the utility of ChatGPT as an AI-assisted decision tool in medical settings for simple conditions, offering timely and precise symptom assessment to enhance patient care.

Differential diagnosis: With the ability to process large datasets, ChatGPT can generate comprehensive differential diagnosis results when a patient’s medical history, symptoms, and physical signs are inputted. In a recent study by Hirosowa et al., the diagnostic accuracy of differential diagnosis lists generated by ChatGPT-3.4 and ChatGPT-4 language models for complex clinical cases was assessed. The rate of correct diagnosis by ChatGPT-4 was comparable to that by physicians within the top 10 and top 5 differential diagnoses with 83% and 81% accuracy, respectively [35,36].

Referral assistance: ChatGPT can help individuals choose appropriate physicians by evaluating their signs and symptoms and suggesting appropriate referrals to general or subspecialty cardiologists. By bridging the gap between patients, physicians, and informational resources, ChatGPT can become a valuable tool for timely diagnosis and improved outcomes and quality of life for individuals with HF [29,37,38].

## 5. Role of ChatGPT in HF Management

Recent studies have demonstrated the beneficial effects of new pharmacotherapies, novel devices, closer monitoring, and treatment adherence in improving survival rates and decreasing readmission rates among patients with chronic HF [6]. Despite the novel advancements in managing HF, challenges remain in understanding the pathophysiology of HFpEF, its phenotypes, and the variable responses of HFpEF and HFrEF to medical, percutaneous, and surgical interventions. In addition, the scarcity of medical resources and perceived economic burden underscores the pressing need for novel and innovative approaches to improve HF management [39]. There is also a critical need for additional research on the effectiveness of emotional and social support on the outcomes of HF management [40]. In this context, we aim to review the potential of the conversational ChatGPT model in the holistic management of HF.

Understanding diagnosis and medical translation: Diagnosis and medical terminology associated with HF can be overwhelming. The generative AI model can explain complex medical contexts clearly by offering relatable examples, interactive Q&A sessions, and simple plain language explanations. For instance, Kozily et al. found that the ChatGPT-3.5 model responded appropriately in a clear, understandable language to questions regarding HF diagnosis, management, and prognosis with 90% accuracy and a high degree of consistency (93%) [41]. This application can contribute to improvement in self-education and foster better communication between patients and healthcare providers [42].

Nutritional and lifestyle recommendations: Dietary and behavioral modifications play a significant part in the overall management of chronic HF [43,44]. However, currently, there are fewer existing dietary strategies proven to improve HF outcomes [43]. ChatGPT models have shown proven effectiveness in providing healthcare recommendations [45]. For instance, Al-Anezi et al., in a quasi-experimental study, found that ChatGPT-3.5 supported health literacy and served as a virtual health coach for chronic disease management in 29 adult patients [43]. In the study, 62% of the adults reported that ChatGPT assisted them in adopting healthy diets, sleep hygiene, and daily exercise goals. Similarly, Dimitriadis et al. explored the accuracy and reproducibility of ChatGPT in answering frequently asked HF questions and found that the language model answered lifestyle and dietary questions with 81% accuracy. It also provided accurate and comprehensive information on nutritional measures, smoking cessation, and alcohol consumption [37].

Medication management: Medication therapy management remains the cornerstone of HF care. The disease complexity and co-morbidities vary widely among individuals with HF, increasing the risk for multiple drug interactions (DIs). ChatGPT, with its ability to integrate large datasets such as medical record databases, can keep track of patients’ co-morbidities, medication regimens, and genetic susceptibilities and provide personalized recommendations. Roosan et al. found that ChatGPT, assessed by two clinical pharmacists, accurately solved DIs and medication adjustment in 39/39 (100%) of cases with variable complexities. ChatGPT could also suggest correct recommendations for alternative medicines in all cases [46]. In an experimental study, Al-Ashwal et al. assessed the effectiveness of various chatbots. They found that they could identify DIs in 225 drug pairs with significant accuracy compared to conventional DI tools [47]. In another study, the language model provided practical and valuable guidance for treatment medication with 66% accuracy [37]. Thus, ChatGPT can be a supportive tool in improving health literacy and minimizing medication adverse events, thereby improving adherence to therapy [48].

Telehealth and remote monitoring: Virtual visits and telemedicine are becoming increasingly common in modern medicine. Incorporating AI into digital health is a potential way of augmenting HF multidisciplinary integrated care [12,49]. ChatGPT language models, by offering a central platform to share patient data and insights, can assist in proving better communication and collaboration among healthcare providers. Alanzi et al. conducted a qualitative approach through focus group discussions involving 54 teleconsultants with varying degrees of experience in ChatGPT. The study identified the positive impact of ChatGPT on 12 themes: informational support, diagnostic assistance, communication, enhancing efficiency, cost and time saving, personalizing care, multilingual support, assisting in medical research, decision-making, documentation, continuing education, and team collaboration [50]. However, ChatGPT generated ineffective responses on legal and ethical issues, misdiagnosis, errors, and limited medical knowledge content [50]. The study highlights the dual aspects of integrating ChatGPT in teleconsultations—improving service delivery while presenting new challenges related to legal and ethical aspects.

Advanced HF care: The global rise in the burden of HF is postulated to increase the incidence of advanced HF, which remains a clinical challenge for both patients and physicians. ML models have demonstrated effectiveness in improving care across the spectrum of advanced HF [12,51]. ChatGPT, with its ability to generate instantaneous personalized recommendations and real-time feedback, can guide advanced HF patients to seek early medical attention and provide education about the benefits and risks of mechanical circulatory support (MCS) systems and heart transplantation. For example, Koh et al. identified that ChatGPT generated appropriate responses to the role of palliative care and the use of left ventricular assist devices in class D HF patients [52]. The language model can assist heart transplantation by improving registry management and care coordination. AI algorithms can facilitate the quick retrieval of critical matching information based on logistical and medical data [53]. ChatGPT can also clarify complex matching algorithms for medical professionals and patients, prompting informed decision-making. Additionally, ChatGPT enhances transplant coordination by delivering customized educational content to patients, thereby improving communication and understanding throughout the transplant process [54,55]. ChatGPT can also provide technical support to physicians troubleshooting MCS and offer surgical assistance and personalized recommendations on immunosuppressive therapy to maximize graft tolerance and minimize infections [54].

Emotional and social support: The current literature shows that individuals with HF with strong emotional and social support have a better quality of life and maintain positive self-care behaviors [56,57]. In an experimental study by Dimitriadis et al., ChatGPT accurately answered vital questions on familial and social assistance of caregivers in the management of HF [37]. The language model also generated responses about community resources and support groups for HF patients [37]. ChatGPT can help individuals express their concerns, share ideas on emotional coping strategies, and motivate patients to follow their treatment plans by utilizing reinforcement learning from human feedback. Elyoseph et al. evaluated ChatGPT’s emotional awareness using the Levels of Emotional Awareness Scale (LEAS). ChatGPT’s responses to 20 scenarios were assessed twice, initially and again a month later. In the first evaluation, ChatGPT outperformed the general population norms on all LEAS scales (Z score 2.84), and its performance improved significantly in the second assessment, nearly reaching the highest possible LEAS score (Z score 4.26) with an accuracy of 97%. Two licensed psychologists confirmed the high accuracy of ChatGPT’s contextually appropriate responses [58]. The usefulness of the ChatGPT language model in reducing feelings of isolation, loneliness, and stress needs to be explored.

**Figure 3:**
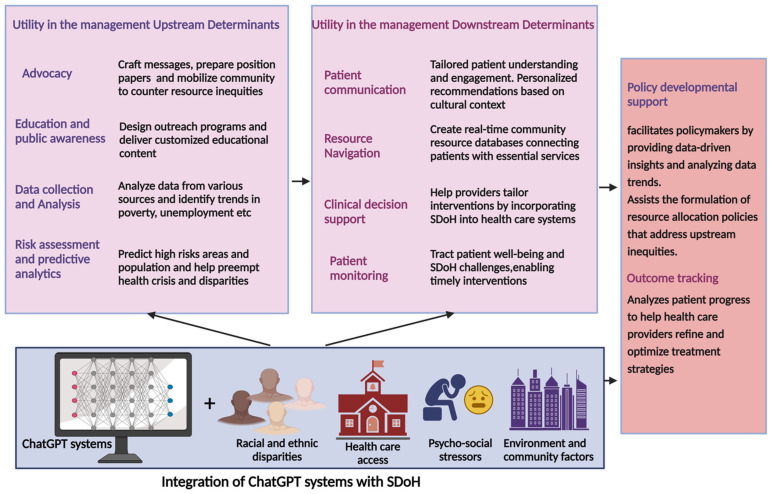
The utilization of ChatGPT in the effective management of SDoH. ChatGPT systems can manage upstream determinants of heart failure by supporting advocacy, education, and policy development, enhancing efforts to address socioeconomic inequities. They also improve patient communication, facilitate access to resources, support clinical decisions, and monitor patient outcomes for downstream determinants.

Social determinants of health (SDoH), encompassing a wide range of social, economic, environmental, and interpersonal factors, play a vital yet under-recognized part in overall HF care [39,59,60]. SDoH are usually divided into two groups: Upstream SDoH, such as disparities in resource distribution, are primarily outside of individual control. Downstream SDoH reflects the downstream effects in clinical settings, highlighting the need for collaborative efforts among healthcare providers and policymakers to mitigate the disparities in HF outcomes [39,61,62]. ChatGPT can improve SDoH education among healthcare professionals, addressing educational gaps and facilitating effective interprofessional collaboration among diverse health teams for coordinated care [63]. It has the potential to streamline the SDoH assessment process using standardized tools like the Accountable Health Communities Social Needs Screening Tool to categorize patient needs and link them to necessary community resources such as housing and food support [64]. In addition, ChatGPT, by analyzing large comprehensive SDoH datasets, can help stratify patients based on their risk of adverse health outcomes. This can enable healthcare providers to prioritize interventions for those most at risk and implement preventative measures tailored to the social contexts of the patients’ lives [65]. Similarly, the language model can also assist healthcare organizations in allocating resources more effectively, ensuring that interventions such as community health programs or educational initiatives are directed where they are most needed. ChatGPT can also extract SDoH data from unstructured electronic health records (EHR) and patient reports [66]. In a recent study, Guevara et al. evaluated the ability of large language models (LLMs), specifically the Flan-T5 model, to identify social determinants of health (SDoH) in electronic health records (EHRs). Flan-T5, a version of Google’s T5 fine-tuned on diverse tasks, demonstrated strong performance with macro F1 scores of 0.71 for any SDoH mention and 0.70 for adverse mentions. The macro F1 score, which balances precision and recall across all categories equally, reflects the model’s robustness in accurately identifying SDoH. Notably, the LLMs identified 93.8% of SDoH mentions in EHRs, compared to only 2% captured by standardized ICD codes, highlighting the superior ability of LLMs to extract meaningful social health data from unstructured text [67]. The addition of artificially generated data by ChatGPT-3.5 significantly improved the performance of smaller models [67]. This is particularly useful for categories like “housing” or “parent”, where only a few real-world examples are available. By providing additional examples for training, synthetic data effectively expands the dataset’s size and diversity, obviating the need to collect more real data. Through ongoing monitoring and feedback mechanisms, ChatGPT can continuously refine SDOH-related strategies and interventions, ensuring they remain effective and responsive to patient needs, enhancing overall patient outcomes in HF management. The utilization of ChatGPT in effectively managing SDoH is shown in Figure 3.

## 6. Research

Applications in Research Design: ChatGPT has demonstrated its utility in research efficiency by automating critical tasks such as hypothesis development, comprehensive literature searches, methodology structuring, and scientific writing [68]. By automating these foundational tasks, ChatGPT allows researchers to focus more on academic productivity and less on the administrative burden of study setup. Wang et al. demonstrated the potential of ChatGPT in generating effective Boolean queries for systematic review literature searches, particularly for rapid reviews, where it achieved higher precision and lower recall compared to current query formulation methods [69]. Similarly, Teperikidis et al. successfully used ChatGPT to support each step of the umbrella review process, including screening by title and abstract, data extraction, study summarization, qualitative synthesis, and risk of bias assessment to assess the causal relationship between proton pump inhibitors and major cardiovascular events [70]. In another interesting commentary, ChatGPT was determined to be a co-author or primary author as it was used to generate 100% of the text efficiently [71].

Data Management and Analysis: ChatGPT integration enhances HF research analysis by data preprocessing, generating reports and visualizations, predictive analytics guidance, data stratification, and automation. For example, Nakaya et al. assessed ChatGPT’s effectiveness in automating bibliometric analysis, specifically classifying virtual reality studies in cardiology. Their findings indicate that ChatGPT correctly classified study abstracts into groups A and B with an accuracy of 97% (sensitivity: 0.98, specificity: 0.96) [72].

**Figure 4:**
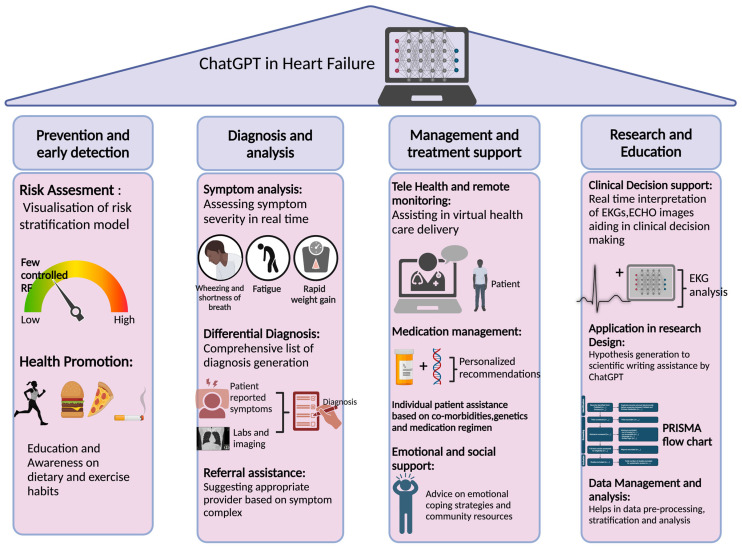
Role of ChatGPT in heart failure management. Multifaceted applications of ChatGPT in heart failure, spanning four key areas: Prevention and Early Detection, Diagnosis and Analysis, Management and Treatment Support, and Research and Education. ChatGPT can assist in risk assessment, health promotion, symptom analysis, referral assistance, telehealth support, personalized medication management, emotional support, and in research activities like hypothesis generation and data analysis. EKG: Electrocardiogram; ECHO: Echocardiogram; PRISMA: Preferred Reporting Items for Systematic Reviews and Meta-Analyses.

Clinical Decision Support: ChatGPT, by analyzing large datasets, can assist healthcare providers in reviewing electrocardiograms (ECG), echocardiograms, and radiographic images. Recently, Olander et al. generated a ChatGPT ECG analysis software focusing on ECG interpretation and analysis [73]. This AI language model uses optical character recognition (OCR) or other image processing techniques to convert an ECG image into a digital waveform, then analyses these waveforms to extract critical features such as P, QRS complex, and T waves and other ECG segments, generating a report based on pattern recognition by ML models. In an experimental study by Fijacko et al., ChatGPT-4 interpreted nine advanced cardiovascular life support ECGs by the American Heart Association with 63% accuracy (17/27). The overall level of correctness of the ECG image by the ChatGPT language model was 78.9% (95% CI: 74.5–83.3) [38]. With the ability to integrate large datasets, ChatGPT generates risk prediction models and targeted drug development [74,75]. We summarize the role of ChatGPT in heart failure management under four broad categories in Figure 4.

## 7. Limitations and Future Scope

AI chatbots like ChatGPT have demonstrated impressive capabilities in different aspects of HF management. However, they are subjected to various unique limitations, and their application in real-world settings is challenging, particularly in complex clinical settings where high-level critical thinking is necessary. In addition, it is important to acknowledge many of the studies reviewed here are preliminary in nature, often characterized by small sample sizes and limited generalizability. The absence of a systematic review process in several of these studies further limits the ability to draw definitive conclusions from the existing data. Despite these limitations, the preliminary studies reviewed provide valuable insights and highlight emerging trends that warrant further investigation [29,33,36]. In this review article, we have broadly divided the practical constraints of ChatGPT application in HF management into three major categories:

### 7.1. Training Data Limitations and Validity

The ChatGPT language model has been trained on a diverse range of parameters. However, the model’s training data only extends to September 2021, and the more advanced ChatGPT-4 was trained only up to January 2023 [20,21], thus preventing ChatGPT from incorporating newer developments and clinical practice guidelines. Additionally, the model was not designed to fully understand the complexity and context of a medical scenario, thereby limiting its application for answering medical questions. The training of the language model on medical data is also tricky due to the sensitive nature of the information and the model’s lack of direct access to EHRs or healthcare databases. ChatGPT has a non-deterministic nature, often producing different responses to identical prompts, raising concerns about the reproducibility and validity of the language model. In addition, responses can be modified by rephrasing prompts and changing the input text, further questioning its applicability in healthcare settings where consistent and accurate information is crucial. For example, Funk et al. found that ChatGPT-3.5 generated varied responses when asked the same medical examination questions in three separate rounds. The accuracy of correct response was quantified at 57.6%, 57.1%, and 58.4% for the first, second, and third rounds, respectively, with a consistency rate of only 44.9% [76].

### 7.2. Accuracy and Bias

The training datasets are not ideal, making the ChatGPT language model inherently susceptible to algorithmic bias and external validity. Kamulegeya et al. found that the ChatGPT model on skin lesion classification, trained predominantly on data from white individuals (90%), had lower diagnostic accuracy in patients of other ethnicities (5–10%) [77]. Additionally, Ineffective or faulty training may generate superficial, inaccurate, and/or incorrect content. Sometimes, the incorrect responses appear plausible from a scientific point of view, introducing hallucination bias. Hallucination bias is a unique problem related to ChatGPT models, where a language model generates output data that is not grounded in facts or does not exist in training data [78]. Addressing this bias involves improving the quality and quantity of training data, fine-tuning the model with accurate supervised content, and creating robust validation tools to check the outputs. Retrieval-Augmented Generation (RAG) is another novel way of enhancing the credibility of the generated responses by incorporating knowledge from real-time external datasets. This is particularly helpful in knowledge-intensive settings where continuous integration of newer updates and reducing inaccuracies/fabrications is necessary. For example, when an individual asks ChatGPT “what are the latest treatment protocols in HF?”, with the help of RAG, it could retrieve the latest guidelines from medical databases and offer an evidence-based generated response like “According to the latest 2022 HF guidelines published in Journal of American College of Cardiology; guideline directed medical therapy for HFrEF now includes four medication classes that include Sodium-glucose cotransporter-2 inhibitors (SGLT2i)” [79,80]. Another common problem with ChatGPT is that it might produce non-original, over-detailed, or excessive content, creating a burden for the user. This can be improved by giving proper prompts and guided feedback (input text), as response generation varies based on prompt construction. For example, instead of asking ChatGPT “Can you tell me about heart failure management?”, a more specific step-by-step prompt such as “Explain the step-by-step process of managing a patient with heart failure with reduced ejection fraction, including diagnosis, treatment and follow-up” can help ensure a more structured and accurate response while minimizing errors.

In addition, it is only logical to question the originality and innovation of the ChatGPT-generated responses. Numerous recent studies have reported the fabrication of medical content and references in biomedical articles by ChatGPT. Bhattacharyya et al. found that of 115 references generated, 47% were fabricated, 46% were authentic but inaccurate, and only 7% were authentic and accurate [81]. Similarly, Gravel et al. examined 59 references and discovered that 69% of them were fabricated. They noted that many of these fabricated references cleverly used names of authors with relevant previous publications, had titles that seemed pertinent, and followed a format typical of credible journals [82]. However, some studies found improvement in fabrication and hallucination bias with advanced versions. Walters et al. showed that among 636 bibliographic citations, ChatGPT-3.5 generated 55% while ChatGPT-4.0 generated only 18% fabricated references [83]. Thus, it is essential to critically evaluate and verify the accuracy of responses before incorporating them into a medical context to ensure the reliability of the information presented. An effective strategy to mitigate fabrication in ChatGPT outputs is to directly prompt the model “Is this information accurate, or is there any fabrication? ” or to request specific URLs or DOIs for citations. In many cases, the model will disclose if the content is fabricated or lacks appropriate references.

### 7.3. Privacy and Ethical Concerns

ChatGPT models come with unique ethical challenges from humanistic, algorithmic, and informational perspectives. The advancement of AI necessitates a clear need for comprehensive ethical guidelines and regulatory frameworks that balance risks and benefits [84]. The main factors to consider include determining responsibility for any harm caused by inappropriate advice from ChatGPT and the storage and processing of sensitive information [85,86]. Open AI explicitly disclaims any accountability for generated texts, making registered users solely responsible for errors [87]. Hence, governing bodies should establish practical guidelines and comprehensive laws for using ChatGPT in clinical practice. In addition, ChatGPT systems have unauthorized access and are susceptible to various cyber threats and vulnerabilities, which can lead to data breaches or manipulation of medical advice [88]. Anonymizing health care information, following strict privacy regulations such as Health Insurance Probability and Accountability Act (HIPPA) guidelines, and preventing reidentification are crucial in handling data privacy [89,90]. To minimize privacy risks, healthcare organizations should enforce stringent security measures, such as encryption and access controls, and establish a data governance framework to ensure compliance and responsible data management [91]. Additionally, integrating ChatGPT systems requires transparency and explainability, mainly due to the critical nature of medical decisions, which requires clear justification to foster trust among providers and patients. AI models in healthcare often act as “black boxes”, with complex internal processes that obscure decision-making and complicate informed consent [92,93]. There is an increasing focus on developing more explainable AI models that comply with ethical and operational standards to address these issues [94].

Despite its limitations, ChatGPT can improve current HF care by offering user-friendly and possibly trustworthy information resources. Nov et al. conducted a survey of 430 US adult patients. They found that the ChatGPT and healthcare provider responses were weakly distinguishable (65.5% vs. 65.1%), and patients’ trust in ChatGPT responses was weakly positive (mean Likert score 3.4/5) [95]. One of the main advantages of ChatGPT is its adaptability. The language model can learn from reinforced human feedback, tailoring its responses to patients’ educational levels. This capability enables it to rephrase general written content, moving beyond the typical one-size-fits-all approach [20,21,48]. ChatGPT systems are readily accessible, and the basic version is available at no cost to the public, making it an indispensable tool in modern-day medicine [20,87]. By integrating advanced analytics, ChatGPT can enhance diagnostic accuracy, optimize treatment plans, and streamline patient monitoring in HF. This leads to more efficient use of healthcare resources, reduces unnecessary hospitalizations, and minimizes costly medical interventions, thereby decreasing healthcare expenditures associated with HF [34,37,45,50]. A quarter of US healthcare costs and more than half of physician burnout stem from administrative burdens such as record keeping, excessive EHR documentation, insurance billing, and prescription management [96,97]. Generative AI models present innovative solutions to these administrative roadblocks, particularly in HF, by developing AI-powered scribing solutions, advanced prior authorization strategies, and integration of ChatGPT technology with EHR vendor systems [98,99]. UC San Diego Health, Madison, Wis.-based UW Health, and Palo Alto, Calif.-based Stanford Health Care recently announced a pilot study on integrating OpenAI’s GPT-4 with Epic’s EHR software. (Epic. Epic Systems Corporation. URL: https://www.epic.com/ (accessed on 12 March 2024)).

This collaboration aims to boost the efficiency and accuracy of clinical communications and data analysis while reducing the administrative burden on clinical providers. The first iteration focuses on the In Basket tool, allowing clinicians to review and personalize generative AI-created automated draft responses for asynchronous patient communication. The second use case leverages Epic’s slicer dicer, a data visualization and analysis tool, to automatically suggest relevant metrics based on the user’s search criteria [100]. Similarly, Boston’s children’s hospital works with Nuance, Dragon Ambient eXperience technology (DAX). In this initiative, generative AI can listen to patient–provider interactions during virtual visits and automatically generate accurate clinical documentation. This process involves the AI capturing spoken language in context, converting it into structured data, and inputting it directly into the EHR, thus minimizing manual data entry [101]. These initiatives are instrumental in HF management, where timely, accurate, and personalized patient–provider interaction is crucial. In addition, monitoring of numerous clinical parameters at once and streamlined data exploration can help optimize GDMT and adjust treatment plans dynamically. The generative AI models can also help identify the correlation/causal relationship between various risk factors and HF treatment efficacy/disease progression, thus enhancing the data-driven clinical decision-making process. In addition, ChatGPT systems can help prioritize clinical tasks based on urgency associated with HF management and provider availability. These systems enhance provider and facility utilization by accurately predicting the duration of clinical visits and leveraging historical data. The appropriate integration of generative AI technologies into day-to-day clinical workflows boosts healthcare efficiency and fosters substantial cost savings.

Integrating ChatGPT models into clinical practice presents several challenges, notably the issues of reproducibility and replicability, often stemming from insufficient data sharing and the limited availability of large, publicly accessible databases. Ethical considerations, the lack of incentives for data sharing, and differences in data formats all contribute to the difficulty of disseminating data across platforms [102]. Furthermore, datasets created through commercial collaborations are frequently restricted from public access, which further impedes the generalizability of ChatGPT models [103]. Publicly available datasets play a critical role in assessing the external validity of these models; the American Heart Association’s Precision Medicine Platform, for example, is a recent initiative aimed at expanding public access to clinical data [104]. Although greater data transparency can enhance generalizability testing, successful implementation of ChatGPT models in clinical practice will require robust validation through large, prospective clinical trials. In response to these challenges, the scientific community has introduced guidelines to improve reporting consistency in clinical trials involving artificial intelligence, including the CONSORT-AI and SPIRIT-AI frameworks [105,106]. These efforts aim to promote more rigorous and transparent evaluations of ML models, ultimately facilitating their integration into clinical workflows.

## 8. Conclusions

This review highlights the role of ChatGPT systems in enhancing HF management. AI-driven approaches, like ChatGPT, have the potential to deepen our understanding of the intricacies of HF care and improve patient-centered care, a cornerstone of modern healthcare. However, the effective integration of ChatGPT language models in daily medical practice faces several challenges, including data validity, ethical concerns, and training limitations. Addressing these issues necessitates refined model training, greater transparency in AI decisions, and robust regulatory frameworks. Furthermore, developing ML tools that are technically adept and attuned to diverse patient needs is crucial, thereby boosting trust and reliability in ChatGPT-supported healthcare. Ultimately, leveraging AI chatbots like ChatGPT in HF management could transform patient outcomes by fostering more personalized and proactive treatment strategies, ensuring that technology enhances rather than replaces the human elements of patient care.

## Figures and Tables

**Table 1:** Milestones toward the evolution of ChatGPT.

Year	AI Model (Dataset)	Estimated Parameters	Additional Value
2018	GPT-1 (BookCorpus).	117 million.	Made a significant shift in how Large Language Models were built.
2019	GPT-2 (WebText).	1.5 billion.	Generated longer and more coherent data text that was difficult to distinguish from human text. Zero-Shot learning capability: generated appropriate responses for text that was not trained.
2020	GPT-3 (Extended WebText).	175 billion.	Generated high-quality natural language loner text with high coherence and realism. Enhanced Zero-Shot learning capabilities. Few-Short learning: generated appropriate answers to text with limited examples. Multi-task learning: ability to perform multiple tasks simultaneously. Real-world applications and greater versatility: chatbot development, language translation, content generation, and code generation. Reduction in training biases: increased diversity in training data and advanced model architecture limited some of the biases present in previous models.
2023	GPT-4.	1.73 trillion.	Generated a large multimodal language model capable of understanding and generating responses to text and images. Best performing GPT model on factuality, steerability, and staying within the boundaries. Reduction in hallucination bias: GPT-4 generated more reliable and accurate responses with reduced occurrence of hallucinations. Better handling of nuanced instructions: GPT-4 performs better with nuanced and complex instruction, understanding more subtle aspects of prompts.

Table 1 summarizes the development of Generative Pre-trained Transformer (GPT) models. GPT-1 started with foundational changes and 117 million parameters. GPT-2 increased to 1.5 billion parameters in 2019, enhancing text coherence and learning capabilities. GPT-3 introduced multitasking in 2020, with 175 billion parameters. The latest, GPT-4, introduced in 2023, features 1.73 trillion parameters, significantly advancing multimodal responses and reducing biases, making it highly relevant for complex applications such as medical research.

## Abbreviations and Acronyms

HF: heart failure; AI: artificial intelligence; ML: machine learning; GPT: Generative Pre-trained Transformer; LLM: large language models; NLP: natural language processing; HFrEF: heart failure with reduced ejection fraction; HFpEF: heart failure with preserved ejection fraction; HFmrEF: heart failure with mildly reduced ejection fraction; GDMT: guideline-directed medical therapy
